# Distribution of the T12 erector spinal muscle plane block in the dorsal region guided by ultrasound

**DOI:** 10.1186/s13018-020-02195-3

**Published:** 2021-01-12

**Authors:** Jin-Feng Zhang, Wei-Wei Zhang, Jia Wang, Hao Guo, Ting Wang

**Affiliations:** Department of Anesthesiology, Shanxi Provincial People’s Hospital, No. 29 Shuangtasi Street, Yingze District, Taiyuan, 030012 China

**Keywords:** Distribution, Spinal muscle, Plane block, Dorsal region, Ultrasound

## Abstract

**Background:**

This study aimed to explore the distribution of the erector spinal muscle plane block of the thoracic 12 vertebral body (T12) in the dorsal region guided by ultrasound.

**Methods:**

A total of 28 patients, who underwent elective lumbar surgery, were enrolled in the present study. These patients were aged between 18 and 65 years, and the American Society of Anesthesiologists (ASA) grade was 1 or 2. The block of the T12 transverse process erector spinal muscle was performed under the guidance of ultrasound, and each side was injected with 25 ml of 0.4% ropivacaine hydrochloride + 2 mg of dexamethasone. The back areas were measured using the cold-warm method (the back area was divided into 11 areas [T7–S1] with the body surface marker). At 10, 20, 30, 40, 50, and 60 min after the drug injection, the effectiveness of the regional block was recorded. The presence of puncture hematoma, local anesthesia drug poisoning, nausea, vomiting, headache, and dizziness after the block was recorded.

**Results:**

The range of the T12 transverse process block was basically fixed at 30 min after the single injection. No pneumothorax, hematoma, or local anesthetic poisoning occurred in any of the patients.

**Conclusion:**

The effective longitudinal plane of the T12 transverse process erector spinal muscle block was mainly distributed in the T9–L5 dorsal cutaneous branches, and the distribution of the block area was safe and stable.

## Background

The erector spinal muscle plane block (ESPB) has been a novel regional block technique in recent years. However, its indications have not been clearly defined [1, 2]. With the development of ultrasound visualization technology and the clear elucidation of the neuroanatomy of the posterior branch of the spinal nerve, the bilateral ESPB has been more and more frequently applied in clinics. However, the anatomy, injection method, diffusion range, dose, and volume of local anesthetics are not clear [3, 4]. ESPB can choose different transverse process levels, and it is easy to control the block range. However, its disadvantage is that there are currently fewer relevant clinical studies. The study has revealed that under the guidance of ultrasound, the analgesic range of the horizontal T12 ESPB could reach the L5 level. Studies have suggested that local anesthetics can be diffused to L2~L3 when the horizontal block position of the erector spine muscle is T7 [1]. Tulgar et al. [2] found that ESPB was performed in the transverse plane of the L4 vertebral body, and the sensory block plane was measured in the range of T12~L4. However, there is no clear report on the safety and stability of the block area distribution. In addition, the clinical application of the vertical spinal muscle block directly at the level of the lumbar spine has caused some orthopedic surgeons to worry about whether the puncture site will cause incision infection. The present study aimed to explore the distribution of the horizontal ESPB of the thoracic 12 vertebral body (T12) in the dorsal region guided by ultrasound and to explore, as far away as possible from the lumbar incision puncture, whether the drug solution can diffuse the effectiveness and stability of the lumbar region, in order to provide a reference for the analgesia of lumbar surgery.

## Materials and methods

### General clinical data

The Ethics Committee of Shanxi Provincial People’s Hospital approved the present study, and the patients or their families provided informed consent. A total of 28 patients (13 male and 15 female) who underwent elective posterior lumbar surgery under general anesthesia were enrolled in the present study. The age of these patients ranged from 18 to 65 years, with an average age of 53.4 ± 9.8 years. The body mass index (BMI) of these patients ranged from 18 to 28 kg/m^2^ (average: 24.5 ± 1.7 kg/m^2^). The American Society of Anesthesiologists (ASA) grade was 1 or 2. These patients had no history of local anesthetics allergy, mental illness, or coagulation dysfunction.

### Surgical methods

After the patient entered the pre-anesthesia room, the venous access was opened. The blood pressure, electrocardiogram (ECG), and blood oxygen saturation (SpO_2_) were monitored. The patient lay in a prone position. Under the guidance of a portable ultrasound device, 0.4% ropivacaine (25 ml) and dexamethasone (2 ml) were injected into the erector spinal muscles of the bilateral T12 transverse processes. At 10, 20, 30, 40, 50, and 60 min after the block, the sites on the back were defined as observation points. The body back was divided into 11 regions, from T7–L5 along the median posterior line. The cold-warm method (75% alcohol) was used to test the block in each area. When the cold-warm sensation was weakened, it was defined as an effective block. At 10, 20, 30, 40, 50, and 60 min after the injection of the drugs, the effectiveness of the regional block was recorded. The fluctuations of the mean arterial pressure (MAP) and heart rate (HR) were maintained to not exceed 20% of the basic level. The changes in vital signs were measured and recorded every 5 min until the patient entered the operating room from the pre-anesthesia room. The presence of puncture hematoma, local anesthesia drug poisoning, nausea, vomiting, shivering, itching, headache, and dizziness after the block was recorded. After entering the operating room, lumbar posterior spinal canal decompression, intervertebral disc removal, bone graft fusion, and internal fixation were performed under general anesthesia. The patient was placed in a prone position. The operating position was located at the lumbar bridge of the operating bed, with thin pillows on both sides of the iliac part. Surgical procedures: midline incision was made in the back. Skin, subcutaneous tissue, and fascia were cut open, and the supraspinous ligament was exposed. The supraspinous ligament was cut open along the midline of the spinous process to reach the bone, and the sacrospinous muscle was stripped to reach the articular process. An automatic retractor was used to expose the lamina. The vertebral lesion body was located with the aid of c-arm. The entry point of each vertebra was determined. The cone was opened to penetrate through to the pedicle entrance cortex. The instrument was opened to break through cancellous bone material. A probe detected whether the pedicle bone pipeline was broken. Bone wax was applied to the tip of the Kirschner wire for positioning and then exiting the Kirschner wire after determining whether the c-arm was accurate. The appropriate length and type of pedicle screw was selected, and a T-wrench was applied to the screw. C-arm fluoroscopy was used to ensure a good screw position. The scissor spinous process was applied to remove the required segment of the spinous process. The lamina needed to decompression segment was bitten by the vertebral plate forceps, and the nerve exfoliator was used to explore the nerve root canal. The posterior longitudinal ligament, annulus fibrosus, and nucleus pulposus were exposed using forceps to remove the intervertebral disc. A connecting rod and bone graft, flat screen, and cross-connection were installed. The incision was rinsed, hemostatic, drainage was placed and sutured.

### Statistics analysis

The data were statistically analyzed by using statistical software SPSS 22.0. Normally distributed measurement data were expressed as mean ± standard deviation (*x* ± SD), and subjected to cluster analysis [5].

## Results

### Block effect

Under the guidance of ultrasound, the block range of the erector spinal muscle of the T12 transverse process was basically fixed, covering the T8–L5 level. The effective block region covered the T9–L5 levels in ≥ 80% of patients and the T8–L5 levels in ≥ 50% and < 80% of patients (Table 1). The effective rate of the block in the different regions was systematically clustered. According to the cluster results and professional knowledge, 11 different regions were divided into three clusters. Among these, the 3rd–9th regions were the first cluster, the 2nd and 10th regions were the second cluster, and the 1st and 11th regions were the third clusters. The comprehensive analysis results of observation time and effective block rate revealed that the first cluster was the area that had a rapid onset of the block. The onset time of the second cluster was slower than that of the first cluster, and the onset time of the third group was the slowest. For the whole block onset time, at 20 min after the injection, the onset of the block was initiated. At 30 min after the injection, the block effect was relatively stable, and within 40–60 min after the injection, there were no significant changes in block effect.

**Table 1:** The effective rate of the block in all regions in patients at different time points after the horizontal erector spinal muscle block of the T12 transverse process (%, *n* = 28)

Block region		After the block					
		10 min	20 min	30 min	40 min	50 min	60 min
1	T7~T8	0	0	3	3	3	3
2	T8~T9	0	25	48.2	50	50	50
3	T9~T10	0	57.1	91	92.8	92.8	92.8
4	T10~T11	11.1	60.7	96.4	96.4	96.4	96.4
5	T11~T12	44.6	69.6	92.8	92.8	92.8	92.8
6	T12~L1	42.8	76.7	87.5	89.2	89.2	89.2
7	L1~L2	42.8	75	87.5	87.5	87.5	87.5
8	L2~L3	0	78.5	87.5	91	91	91
9	L3~L4	0	51.7	83.9	85.7	85.7	85.7
10	L4~L5	0	10.7	73.2	82.1	82.1	82.1
11	L5~S1	0	0	12.5	14.2	14.2	14.2

A further cluster analysis was performed on the onset rate of the block in different time intervals, and the overall onset rate was gradually increased with time. The clustering was conducted according to the improvement degree of the effective rate in different time intervals. The regions were divided into two clusters. The first cluster was the 1st, 2nd, 10th, and 11th regions, while the second cluster was the 3rd–9th regions. The first cluster had a slowly increased block rate, while the second cluster had a rapid onset within 20 min and subsequently became stable.

### Adverse reactions

All 28 patients successfully completed the bilateral ESPB under ultrasound guidance. No local hematoma, pneumothorax, infection, nerve injury, or other complications occurred, and no adverse reactions, such as nausea, vomiting, dizziness, or drowsiness, were observed. One patient had transient mild hypotension and mild bradycardia, which was relieved after intravenous injection of 0.5 mg of atropine.

## Discussion

Posterior lumbar surgery has a large trauma range. This operation can strip and pull the blocked paravertebral regions, which can cause moderate to severe postoperative pain and delay postoperative recovery. The inflammatory reactions caused by surgical trauma may lead to peripheral and central sensitization, making the postoperative pain more difficult to control [6]. Present studies have reported that the ESPB should be performed at the level of the lumbar vertebrae. However, since the puncture site is close to the incision site, this has caused orthopedic doctors to worry about incision infection after lumbar surgery [7, 8]. Therefore, in the present study, the probe slid apart from the midline by approximately 2.5–3.0 cm while paralleled to the long axis to find the T12 transverse process. The puncture point was the point vertical to the transverse process of T12 and approximately 3 cm horizontally apart from the head of the long axis to make it as far as possible from the incision [9].

A related study revealed that the concentration of local anesthetic is an important factor in the onset time of the nerve block. The higher the concentration was, the faster the onset became [10]. In clinics, the commonly used concentration of ropivacaine is 0.375–0.500%. A total of 50 ml of 0.4% ropivacaine was bilaterally used to help the concentration and volume reach a proper ratio to achieve the expected effect in the present study. This approach achieves the clinical effect and does not affect the safety of the patient [11]. In the present study, an ultrasound-guided bilateral ESPB was conducted. Under dynamic direct vision, local anesthetics were accurately injected into the deep surface of the erector spinal muscle using the in-plane technique, and the diffusion of the local anesthetic was observed. The results revealed that the effect of the block was good.

The present study results revealed that the block effect was relatively stable at 30 min after the horizontal T12 erector spinal muscle block. The range of the block was within the T9–L5 levels. The effective block area was within the T9–L5 levels in ≥ 80% of patients, while the effective block area was within the T8–L5 levels in ≥ 50% and < 80% of patients.

Cluster analysis is a statistical method in which the subjects are divided into different subtypes based on their difference in similarity degree. The similarity between different clusters is observed and explored. In the present study, the distribution of the effective block area of the erector spinal muscle block of the T12 transverse process in the lumbar back block was observed. Cluster analysis was used to analyze and summarize the block effect and onset rate to determine the internal connection of the different block areas. In the present study, the effective rate of the block in different regions was systematically clustered, and the results revealed that 20 min is the rapid onset period of the block. In terms of the whole block onset time, the onset of the block started at 10–20 min after the injection of drugs, and at 30 min after the injection of drugs, the block effect stabilized. There were some differences in the onset rate and effective block rate of the erector spinal muscle block of the T12 transverse process in different regions. The erector spinal muscle covers the whole of the back, and the deep layer of thoracolumbar fascia between the erector spinal muscle and transverse process of the lumbar vertebrae extends from the thoracic vertebrae to the lumbar vertebrae. Therefore, this provides an anatomical basis for the diffusion of local anesthetics in the direction of the head and tail. A study considered that the main mechanism of anesthetics might be the block of the posterior branch of the spinal nerve, which diffuses in the fascial space [12]. In the present study, the liquid medicine diffused to the periphery with the injection point at the horizontal T12 transverse process as the center. In addition, since the present study chose to puncture the needle at 45–60° in the plane, the tip of the needle was paralleled to the caudal side of the spine to inject the liquid medicine, and the liquid medicine spread from the proximal end to the distal end. This shows that the closer the area to the center point, the earlier the spinal nerve contacts with the drugs, and the faster the drug works, while on the contrary, the slower the drug works. The difference in effective rate was most likely because the injected liquid medicine in the thoracic region flowed to the thoracic and lumbar segments, and the anatomical paths of the thoracic and lumbar segments, through which the liquid medicine acted differently on the spinal nerve [13].

In the case of the ESPB under the guidance of ultrasound, the ultrasound image of the transverse process could easily be identified, and there were no significant blood vessels, nerves, or other organs on the transverse process. Therefore, the ESPB can significantly reduce the risk of adverse events, such as hematoma, nerve injury, pneumothorax, and block failure. In addition, blood was not observed during the withdrawal of the needle. Local anesthetics were injected into the deep surface of the erector spinal muscle. The separation of erector spinal muscle from the surface of the transverse process was observed to ensure that the injection site of the local anesthetic was correct and prevent local anesthetic poisoning. The present study revealed that mild hypotension occurred in one patient. In the literature, the view of these paraaxonal nerve blocks was that these blocking techniques not only completely cover the distribution area of cutaneous branches but also block the sympathetic nerve [14, 15].

There are several limitations in the present study. Due to the influence of objective factors, the sample size of this study is limited, which needs to be studied in large sample size in the future; secondly, this study is a descriptive study, and there is no control group, and the control group will be added in the future study for further demonstration.

In summary, in the present study, 25 ml of 0.4% ropivacaine hydrochloride and 2 mg of dexamethasone were used for the ultrasound-guided horizontal erector spinal muscle block of the T12 transverse process, and the block range was from T9 to L5 within 30 min after the injection of the drugs. The effect of the block was good. Therefore, this technique can be used for incision analgesia in this range after lumbar surgery. No pneumothorax, hematoma, or local anesthetic poisoning occurred in any of the patients in the present study. This technique is fairly safe. Further studies can be conducted to explore the effect of the erector spinal muscle plane block on postoperative analgesia and long-term prognosis.

## Data Availability

The datasets generated and/or analyzed during the current study are not publicly available due to the lack of an online platform but are available from the corresponding author on reasonable request.

## Abbreviations

ASA: American Society of Anesthesiologists
ECG: Electrocardiogram
MAP: Mean arterial pressure
HR: Heart rate
